# Supplementary material for: Photoprotective Effects of Processed Ginseng Leaf Administration against UVB-Induced Skin Damage in Hairless Mice
Source: Molecules. 2023 Sep 21;28(18):6734. doi: 10.3390/molecules28186734 (PMC10534821; doi:10.3390/molecules28186734)
Supplement: Supplementary file 1 [file molecules-28-06734-s001.zip › molecules-2565407-supplementary.pdf]

Figure S1.

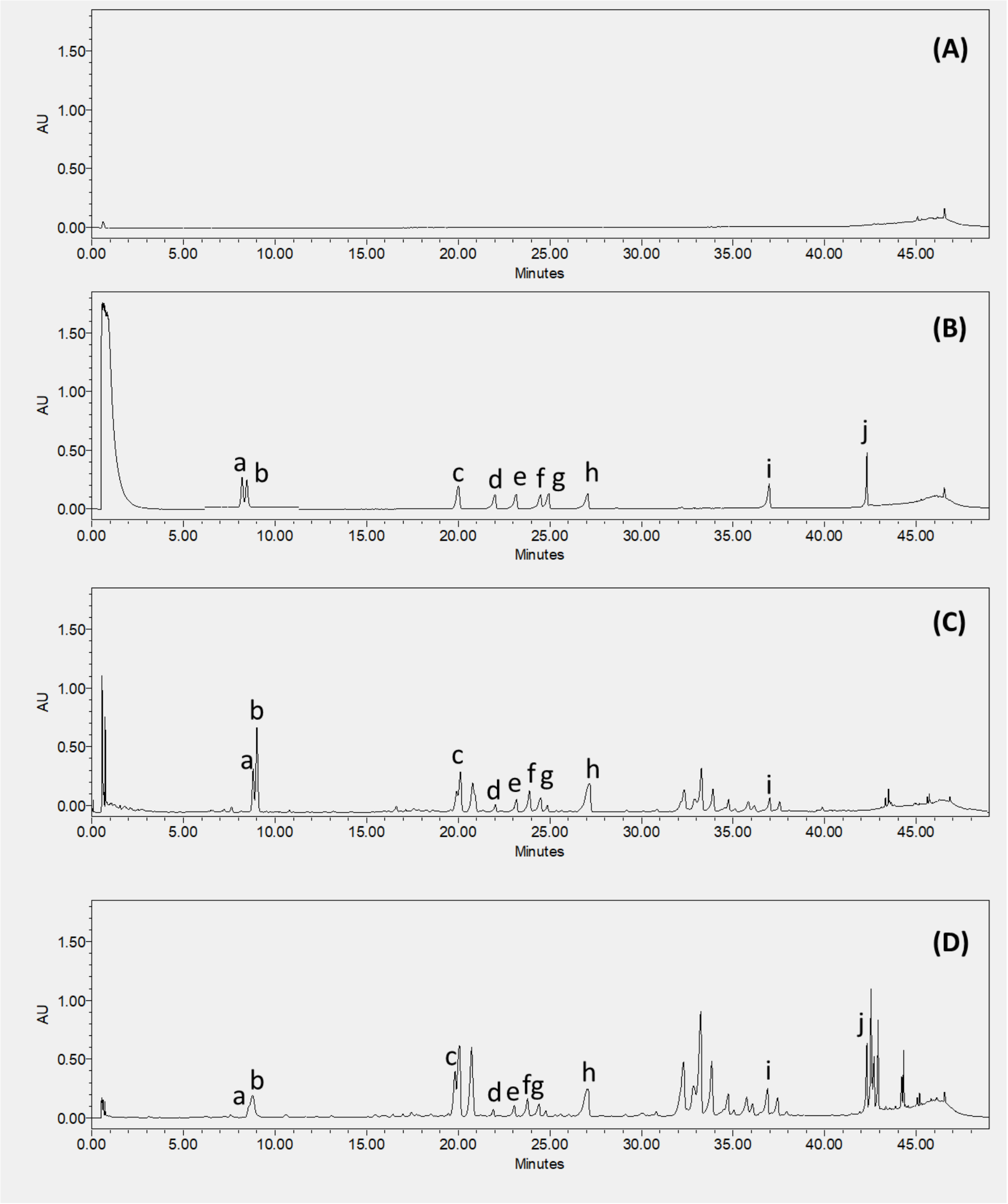

Figure S1. Representative chromatogram of ginseng leaf and processed ginseng leaf extract. (A) blank; (B) reference standard solution, (C) Ginseng leaf extract, (D) Processed ginseng leaf extract. (a) ginsenoside Rg1; (b) ginsenoside Re; (c) Ginsenoside Rg2; (d) Ginsenoside Rb1; (e) Ginsenoside Rc; (f) Ginsenoside Rb2; (g) Ginsenoside Rb3; (h) Ginsenoside Rd; (i) Ginsenoside Rg3; (j) Ginsenoside Rk1.
